# Evaluation of the Multivalent Immunoprotective Effects of Protein, DNA, and IgY Vaccines Against *Vibrio fluvialis* Outer Membrane Protein VF14355 in *Carassius auratus*

**DOI:** 10.3390/ijms26073379

**Published:** 2025-04-04

**Authors:** Huihui Xiao, Jing Chen, Pan Cui, Xixian Che, Xiaoqing Wu, Juan Lu, Guoping Zhu, Yong Liu, Xiang Liu

**Affiliations:** 1 Anhui Province Key Laboratory of Embryo Development and Reproductive Regulation, Fuyang Normal University, Fuyang 236041, China; 22211302@stu.fynu.edu.cn (H.X.); 23211308@stu.fynu.edu.cn (J.C.); 23211320@stu.fynu.edu.cn (P.C.); 2024211305@stu.fynu.edu.cn (X.C.); 201806024@fynu.edu.cn (X.W.); 2 Rural Revitalization Collaborative Technology Service Center of Anhui Province, Fuyang Normal University, Fuyang 236041, China; 200107024@fynu.edu.cn; 3 Anhui Provincial Key Laboratory of Molecular Enzymology and Mechanism of Major Metabolic Diseases, Auhui Provincial Engineering Research Centre for Molecular Detection and Diagnostics, College of Life Sciences, Anhui Normal University, Wuhu 241000, China; gpz1996@yahoo.com

**Keywords:** *Vibrio fluvialis*, multivalent vaccine, protein vaccine, IgY, DNA vaccine

## Abstract

Vaccination is widely recognized as an effective strategy for preventing various bacterial and viral diseases. In this study, protein, DNA, and egg yolk antibody (IgY) vaccines targeting the outer membrane protein VF14355 of *Vibrio fluvialis* (*V. fluvialis*) were administered to goldfish (*Carassius auratus*, *C. auratus*) subsequently challenged with *V. fluvialis* and *Aeromonas hydrophila* (*A. hydrophila*). The immune efficacy of the three VF14355 vaccines was evaluated through their immune activities, protective rates, anti-inflammatory and antioxidant effects, histopathology, and immunofluorescence, and the results indicated that the protective rates in the three immunized groups were significantly higher than those in the control group; furthermore, the number of kidney bacteria was significantly reduced in the immunized group compared to the control group. The ELISA results demonstrated an in vitro interaction between the bacteria and *C. auratus* serum. The plasma phagocytosis index and phagocytosis percentage were significantly increased in *C. auratus*, and their serum immune factor levels, including those of acid phosphatase (ACP), alkaline phosphatase (AKP), and lysozyme (LZM), were increased, while those of serum antioxidant factors, such as superoxide dismutase (SOD), catalase (CAT), and malondialdehyde (MDA), were reduced in the immunized group; notably, the expression levels of inflammatory factors were also diminished in the immunized groups. Histopathological analyses further revealed that the organ structures of the immunized group remained intact, and immunofluorescence tests indicated significant reductions in apoptosis factor p53 and DNA damage factor γH2A.X in kidney tissues. Therefore, the protein, DNA, and IgY vaccines of VF14355 demonstrate the potential to confer resistance against various bacterial infections, positioning them as promising multivalent vaccine candidates for aquaculture.

## 1. Introduction

Aquaculture is a significant agricultural industry [1]; as the scale of breeding continues to expand, the occurrence and prevalence of bacterial diseases are becoming increasingly severe [2], resulting in substantial economic losses for the aquacultural sector [3]. Key pathogenic bacteria affecting aquaculture include *Aeromonas hydrophila* (*A. hydrophila*), *Vibrio fluvialis* (*V. fluvialis*), *Pseudomonas fluorescens* (*P. fluorescens*), *Edwardsiella tarda* (*E*. *tarda*), and *V. parahaemolyticus* [4]. The impact of *V. fluvialis* on aquaculture warrants particular attention; its primary detrimental effects are as follows: Firstly, *V. fluvialis* is a pathogenic bacterium affecting various aquatic animals, including commonly farmed fish such as flounder (*Paralichthys olivaceus*), crucian carp (*Carassius carassius*), and grass carp (*Ctenopharyngodon idella*), potentially leading to fish sepsis, impetigo, and other diseases and resulting in significant economic losses [5]; additionally, it can cause digestive tract inflammation and nutrient absorption disorders in shrimp and other aquatic animals, hindering their normal growth and potentially leading to mortality [6]. Secondly, during its reproduction process, *V. fluvialis* secretes substantial amounts of organic matter, which degrades water quality and increases breeding costs [7]. Furthermore, infected aquatic animals excrete large quantities of waste, exacerbating the pollution of the breeding environment [8]. Notably, *V. fluvialis* is a zoonotic pathogen that can infect both animals and humans, potentially causing gastroenteritis or severe watery diarrhea in humans [9]. Consequently, controlling *V. fluvialis* infection has become a critical issue in maintaining fish health within aquaculture.

Currently, the control of *V. fluvialis* primarily relies on antibiotic treatment; however, this approach has notable shortcomings in the management of fish diseases, including the potential for residue accumulation, the development of bacterial resistance, and environmental pollution [10,11]. Additionally, incorporating Chinese herbs, vitamins, immune enhancers, and probiotics into fish feed can bolster fish immunity, aiding in the prevention of bacterial diseases, however, this method is often slow-acting and costly [12,13]. Thus, novel drugs to control bacteria in aquaculture must be developed.

In recent years, bacterial vaccines have garnered significant attention from researchers due to their lack of need for reliance on antibiotics, absence of toxic side effects, and non-persistence in aquacultural environments [14,15]. The commonly used vaccines in aquaculture include live attenuated vaccines, inactivated vaccines, protein subunit vaccines, DNA vaccines, and mRNA vaccines [16], among which inactivated vaccines are widely utilized, with approximately three such vaccines reportedly licensed in the United States [17], including the *Edwardsiella* vaccine for catfish to prevent enterosepsis, the *Arthrobacter* vaccine for salmon to combat bacterial kidney disease (BKD), and the *Flavobacterium* vaccine for catfish to prevent columnaris infection [18]. Attenuated vaccines have also been successfully implemented in aquaculture [19]; currently, four modified live attenuated vaccines are approved in the United States for the prevention of three bacterial diseases—catfish enterosepsis, bacterial nephropathy, and columnaris disease—as well as in Israel for a viral disease affecting carp, koi herpesvirus [20]. However, the development of protein subunit vaccines for aquatic applications remains slow; therefore, there is a pressing need to develop new vaccines that can effectively prevent and control pathogenic bacteria in aquaculture [21].

Outer membrane proteins (OMPs) exhibit significant immunogenicity and have garnered considerable attention in the development of protein-based vaccines [22,23]. He et al. [24] developed a recombinant protein subunit vaccine targeting receptor-binding domain (PS-RBD), and was utilized to immunize rhesus monkeys, followed by exposure to the Beta variant (B.1.351) of COVID-19. The results indicated that, after the challenge, the viral loads in both the upper and lower respiratory tracts were reduced, pathological changes were less severe, and inflammatory cytokine levels in lung tissue were diminished, with the findings suggesting that PS-RBD offers effective protection against the COVID-19 Beta variant in the rhesus monkey model. Additionally, Howlader et al. [25] formulated a candidate subunit vaccine named ExlA/L-PaF/BECC/ME, which was administered to older mice prior to infection with *P. aeruginosa*, whereafter the immunized mice demonstrated T cell-mediated adaptive responses, elicited anti-*P. aeruginosa* immune responses, and exhibited reduced inflammatory reactions. Consequently, this vaccine shows promise for protecting older adults against *P. aeruginosa* infections. Furthermore, Sun et al. [26] constructed the outer membrane protein ExbB from *Pseudomonas fluorescens* and assessed its potential for passive immune protection; their findings revealed that ExbB could achieve a protection rate of 54% against *P. fluorescens*. However, most of the research on OMP vaccines is still in the laboratory development stage, and no products have entered the market. Furthermore, current research on vaccines targeting *V. fluvialis* OMPs remains limited, particularly regarding the development of multivalent OMP vaccines against multiple bacterial infections; therefore, further investigations are warranted to create multivalent vaccines against bacterial infection.

We commenced this study by investigating the *V. fluvialis* ATCC33809 outer membrane protein VF14355, testing both the active immunity vaccine and the DNA vaccine derived from VF14355, and preparing a passive immunity vaccine using VF14355 IgY sourced from eggs. The three vaccines were administered to *C. auratus*, which were subsequently challenged with *V. fluvialis* and *A. hydrophila*. The immunogenicity of the vaccines was assessed through analyses of immune activity, protection rates, and anti-inflammatory and antioxidant effects, as well as histopathology and immunofluorescence (Figure S1). The findings of this research establish a foundational basis for the development of vaccines in aquaculture.

## 2. Results

### 2.1. Immune Protective Rates of the Protein, IgY, and DNA Vaccines of VF14355

**Figure 1:**
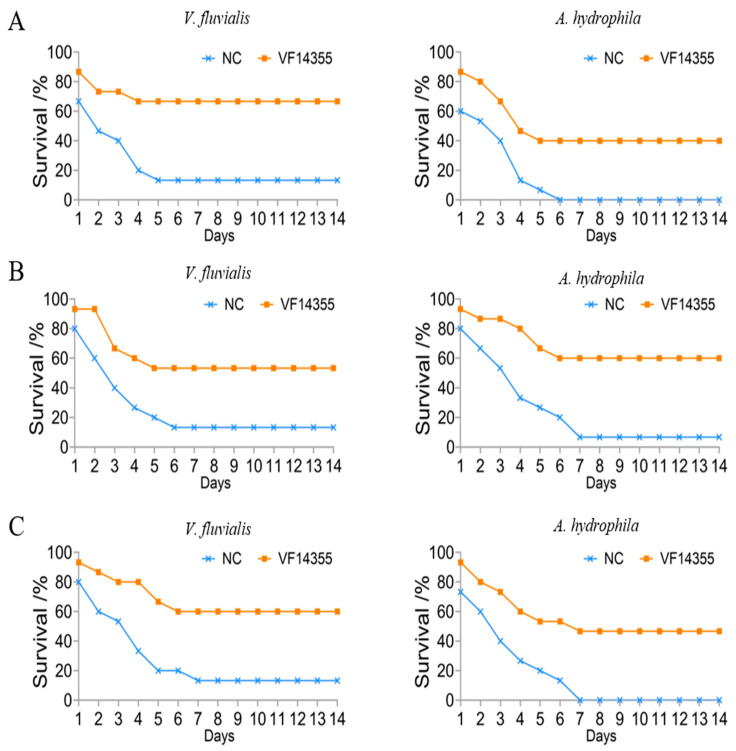
Survival rate of *C. auratus*. (**A**–**C**) The protein, IgY, and DNA vaccines of VF14355.

To evaluate the immune protective rates of the three vaccines, *C. auratus* was immunized with the three vaccines and challenged to *V. fluvialis* or *A. hydrophila*. The challenge experiments demonstrated that, following exposure to *V. fluvialis* and *A. hydrophila*, *C. auratus* exhibited reduced swimming activity, epidermal hemorrhage, abdominal swelling, and significant mortality. The mortality rate stabilized after seven days in the three vaccines (Figure 1). Compared with the nature control group (NC) receiving normal saline, the relative percent survival (RPS) of protein, IgY and DNA vaccines of VF14355 protein against *V. fluvialis* was 61.54% (*p* < 0.01), 46.15% (*p* < 0.01), and 53.85% (*p* < 0.01), respectively; meanwhile, for the three vaccines against *A. hydrophila,* it was 40% (*p* < 0.01), 64.29% (*p* < 0.01), and 46.67% (*p* < 0.01), respectively. Thus, the three vaccines have the immune protective rates to resist *V. fluvialis* and *A. hydrophila* in *C. auratus*, and there is no significant difference among them.

### 2.2. Detection of the Number of Bacteria in C. auratus Kidneys

**Figure 2:**
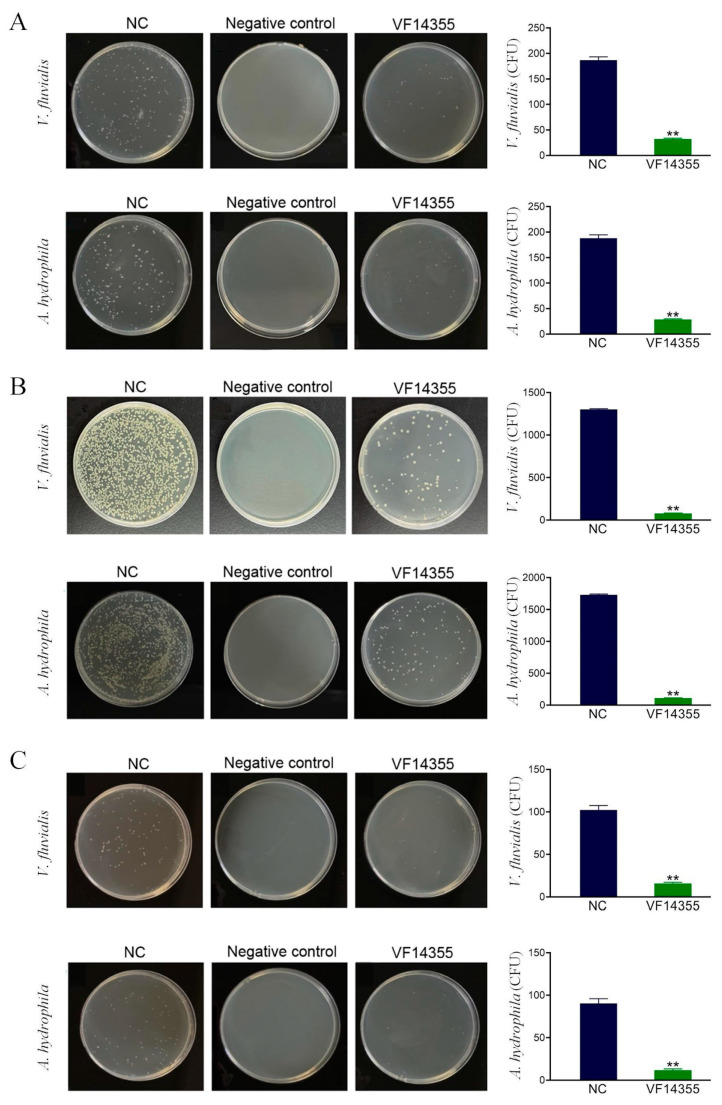
Kidney bacteria count after challenging with *V. fluvialis* and *A. hydrophila*. (**A**–**C**) The protein, IgY, and DNA vaccines of VF14355. Compared with the control group (NC), ** *p* < 0.01. The number of bacteria in *C. auratus* kidneys reduced (*p* < 0.01) in the groups of vaccine immunization. NC refers to the fish receiving normal saline as the nature control group, and negative control refers to the kidneys of fish without attacking bacteria.

To assess the ability of the three vaccines to eradicate bacteria, the Luria–Bertani (LB) medium was coated with homogenates of *C. auratus* kidney tissues for bacterial colony analysis. Bacterial plating results indicated that the number of bacteria in the kidneys of *C. auratus* immunized with the protein, IgY, and DNA vaccines of VF14355 protein was significantly reduced (*p* < 0.01), compared to that in the control group, following challenges with *V. fluvialis* and *A. hydrophila* (Figure 2), suggesting that the three-vaccine immunization effectively inhibits bacterial infection in kidney tissue.

### 2.3. Detection of Plasma Leukocyte Phagocytosis in C. auratus

**Figure 3:**
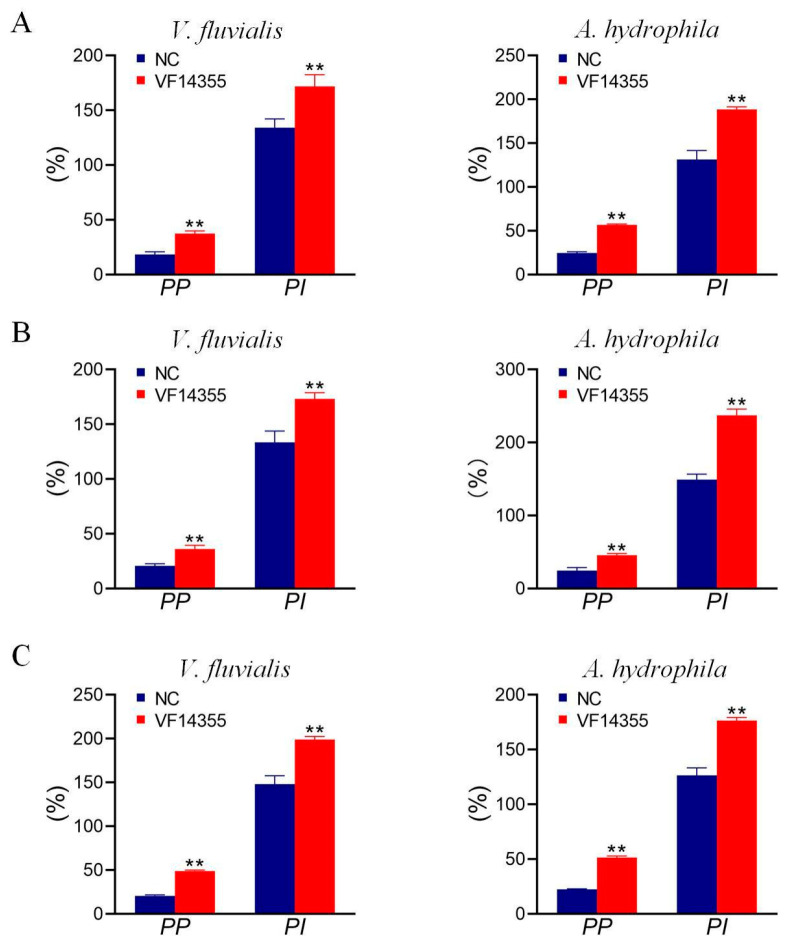
Plasma leukocyte phagocytosis activity in *C. auratus*. (**A**–**C**) The protein, IgY, and DNA vaccines of VF14355. Compared with the control group, ** *p* < 0.01. *PI* and *PP* elevated (*p* < 0.01) in the groups of vaccine immunization.

To assess white blood cell (leukocyte) phagocytosis, *C. auratus* were immunized with the protein, IgY, and DNA vaccines of VF14355 protein and challenged with *V. fluvialis* or *A. hydrophila*. The cell phagocytosis experiment revealed that the leukocyte phagocytosis index (*PI*) and phagocytosis percentage (*PP*) in the plasma of *C. auratus* specimens were significantly elevated (*p* < 0.01), compared to the control specimens (Figure 3), indicating that immunization with the three vaccines activate the phagocytic activity of plasma leukocytes in *C. auratus*.

### 2.4. Expression of Immune Factors

To verify the immune activation effect, the protein and DNA vaccines of VF14355 were immunized to *C. auratus* and laying hen, respectively. The detection of immune factors showed that the levels of acid phosphatase (ACP), alkaline phosphatase (AKP), and lysozyme (LZM) in the serum of *C. auratus* and laying hen were significantly elevated (*p* < 0.01) in the groups of the protein, IgY and DNA vaccines of VF14355 (Figure S2), suggesting that the three vaccines could activate non-specific immunity.

### 2.5. Interaction Detection In Vitro

**Figure 4:**
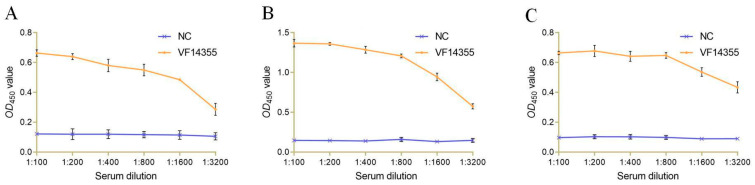
Mutual in vitro recognition between *C. auratus* serum, IgY antibody, and *V. fluvialis*. (**A**–**C**) The protein, IgY, and DNA vaccines of VF14355.

To assess the recognition of serum and bacteria in vitro, the protein, IgY and DNA vaccines of VF14355 were immunized to *C. auratus* and challenged with *V. fluvialis*, and *C. auratus* serum was obtained. The ELISA results indicated that the *C. auratus* serum could bind to *V. fluvialis*, and the absorbance decreased with increasing antibody dilution (Figure 4), suggesting that *C. auratus* serum could recognize with *V. fluvialis* in vitro.

### 2.6. The Titer and Specificity of VF14355 IgY Antibody

To evaluate the titer and specificity of VF14355 IgY antibody, a laying hen was immunized with VF14355 protein, and IgY antibody was obtained. The Western blotting results showed that VF14355 IgY antibody was specifically combined with VF14355 protein. VF14355 stimulated the laying hen to produce a specific IgY antibody with a titer of 1:102,400 (Figure S3).

### 2.7. Detection of Antioxidant-Related Factors in C. auratus Serum

**Figure 5:**
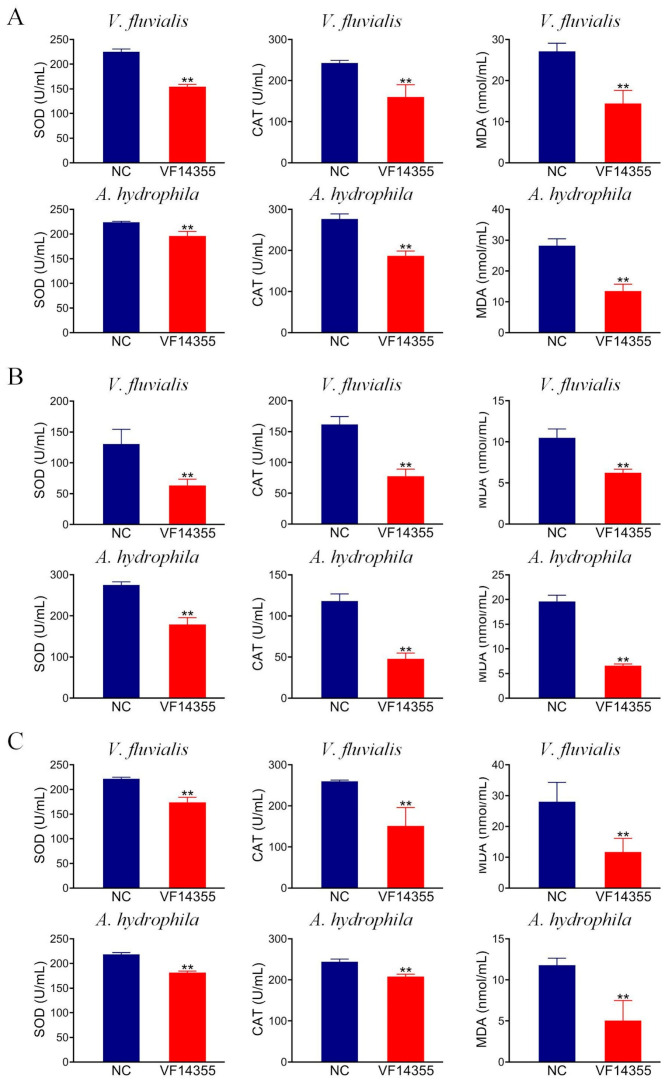
The expression levels of antioxidation-related factors in *C. auratus* serum. (**A**–**C**) The protein, IgY, and DNA vaccines of VF14355. Compared with the control group, ** *p* < 0.01. Antioxidant-related factors (SOD, CAT, and MDA) decreased (*p* < 0.01) in the groups of vaccine immunization.

To assess the effect of the protein, IgY, and DNA vaccines of VF14355 on the antioxidant effects in *C. auratus*, serum was collected after *C. auratus* was immunized with the three vaccines and challenged to *V. fluvialis* or *A. hydrophila*. The results of the antioxidant factor analysis revealed that compared to the control group, the three vaccines exhibited a reduction in most antioxidant-related factors of superoxide dismutase (SOD), catalase (CAT), and malondialdehyde (MDA) in *C. auratus* sera (*p* < 0.01) (Figure 5), suggesting that the three vaccines could mitigate the *V. fluvialis* and *A. hydrophila* infection of *C. auratus* to a certain extent.

### 2.8. mRNA Expression of Inflammation Factors in C. auratus

**Figure 6:**
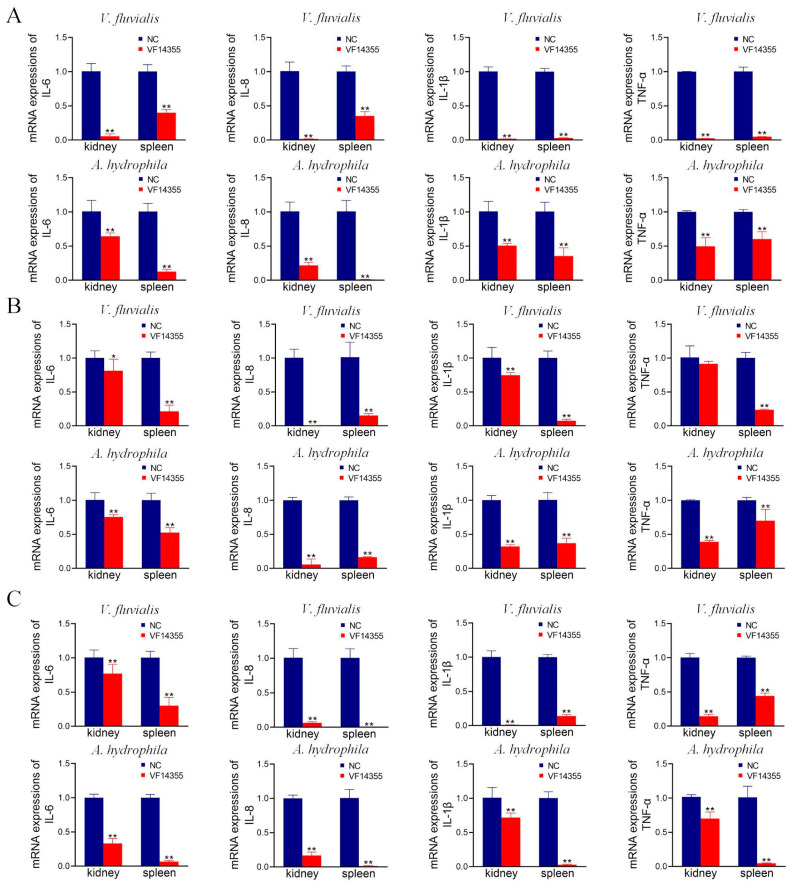
Inflammatory factor mRNA expression levels. (**A**–**C**) The protein, IgY, and DNA vaccines of VF14355. Compared with the control group, * *p* < 0.05 and ** *p* < 0.01. The mRNA expression of IL-6, IL-8, TNF-α, and IL-1β decreased (*p* < 0.05) in the groups of vaccine immunization.

To assess the effect of the three vaccines on anti-inflammatory activities, *C. auratus* was immunized with the three vaccines and challenged to *V. fluvialis* or *A. hydrophila*. The results of the inflammatory factor analysis demonstrated that in comparison to the control group, the mRNA expression levels of IL-6, IL-8, TNF-α, and IL-1β in the kidneys and spleens of the immunization groups of the protein, IgY, and DNA vaccines of VF14355 were significantly reduced (*p* < 0.05) (Figure 6), indicating that the three vaccines could diminish the inflammatory response in *C. auratus* induced by *V. fluvialis* and *A. hydrophila*.

### 2.9. Histopathological Morphological Observation of C. auratus

**Figure 7:**
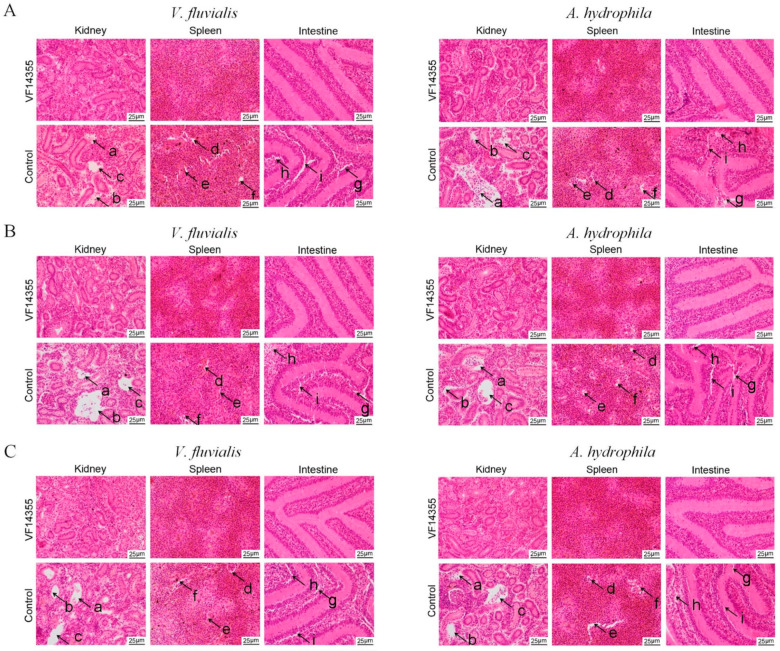
Histopathological kidney, spleen, and intestinal sections of *C. auratus*. (**A**–**C**) represent the protein, IgY and DNA vaccines of VF14355. a–c Indicate cell apoptosis, glomerular degeneration, and tubular degeneration of kidney in the control group, respectively. d–f Indicate cell apoptosis, decreased cell density, and incomplete structure of spleen in the control group, respectively. g–i Indicate cell apoptosis, mucosal lamina propria atrophy, and incomplete structure of intestine in the control group, respectively.

To assess the protective activities of the three vaccines on *C. auratus* visceral structures, kidneys, spleens, and intestines were collected after *C. auratus* were immunized with the three vaccines and challenged to *V. fluvialis* or *A. hydrophila* for histopathological analyses. Histopathological section results indicated that the kidney tissue structures in the control group were loose and incomplete, exhibiting parenchymal damage, severe cell vacuolization, and cell apoptosis. Similarly, spleen tissue was also incomplete, with reduced cell density and occurrences of cell apoptosis; furthermore, the intestinal mucosal lamina propria showed atrophy, and the villous structure was collapsed. In contrast, the immunization groups of the protein, IgY, and DNA vaccines of VF14355 displayed intact and clearly defined structures in the kidney, spleen, and intestine (Figure 7), suggesting that the three vaccines could preserve the integrity of the internal organs to resist the *V. fluvialis* and *A. hydrophila* infections in *C. auratus*.

### 2.10. Immunofluorescence Analysis of C. auratus Kidney

**Figure 8:**
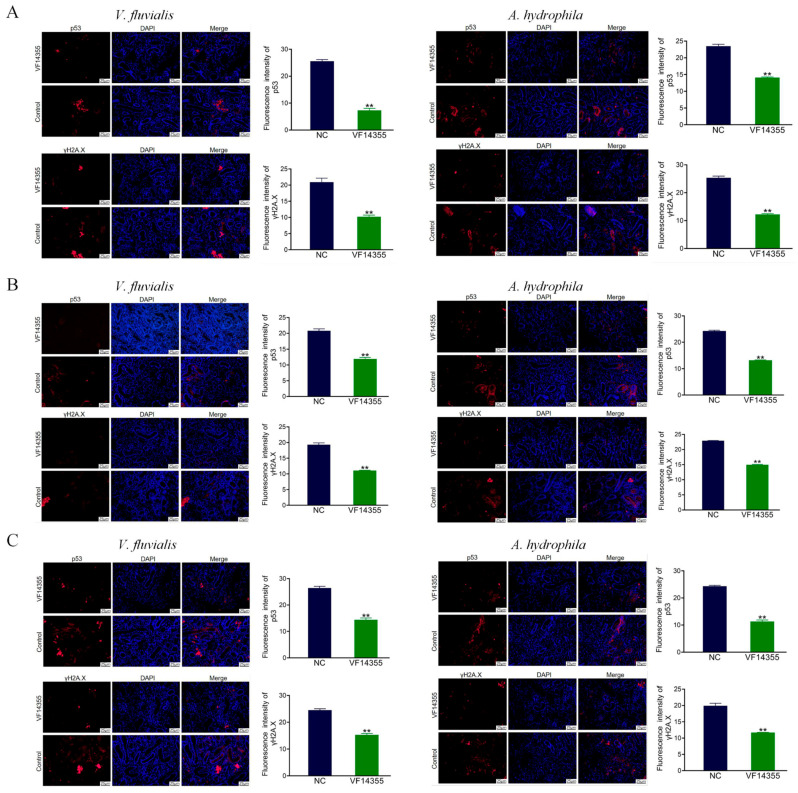
Immunofluorescence analysis of renal p53 and γH2A.X in *C. auratus*. (**A**–**C**) The protein, IgY, and DNA vaccines of VF14355. Compared with the control group, ** *p* < 0.01. The expression levels of p53 and γH2A.X decreased (*p* < 0.01) in the groups of vaccine immunization. Red fluorescence denotes the expression levels of p53 and γH2A.X, while blue denotes a DAPI stained nucleus.

To evaluate the protective activities of the three vaccines on visceral cell function, *C. auratus* was immunized with the three vaccines and challenged to bacteria. Red fluorescence denotes the expression levels of p53 and γH2A.X, while blue denotes a 4′,6-diamidino-2-phenylindole (DAPI) stained nucleus. Immunofluorescence results demonstrated that, compared to the control group, the expression levels of p53 and γH2A.X in the immunization groups of protein, IgY, and DNA vaccines of VF14355 were decreased (*p* < 0.01) (Figure 8), suggesting that the three vaccines could reduce apoptosis and DNA damage in kidney cells to resist the *V. fluvialis* and *A. hydrophila* infections in *C. auratus*.

## 3. Discussion

Active immunity refers to the body’s capacity to generate both humoral and cellular immunity through vaccination, enabling the development of immunity and disease resistance [27]. Common types of active immunity vaccines include inactivated vaccines, live attenuated vaccines, and subunit vaccines. Currently, inactivated and live attenuated vaccines are extensively utilized in aquaculture [28]. However, the practical application of protein subunit vaccines remains limited, although numerous studies have investigated subunit vaccines. In the current study, the VF14355 protein vaccine was utilized to actively immunize *C. auratus* against *V. fluvialis* and *A. hydrophila*. The protection rate in the VF14355-immunized group was significantly higher than that of the control group (*p* < 0.01). A bacterial coating test also revealed a significant reduction in the number of kidney bacteria in the immunized group (*p* < 0.01). Additionally, it was observed that both the plasma phagocytosis index and the phagocytosis percentage of *C. auratus* were significantly increased (*p* < 0.01) and the levels of immune factors ACP, AKP, and LZM were also significantly elevated (*p* < 0.01). An ELISA test was conducted to detect specific indicators in *C. auratus* serum, revealing an in vitro interaction between the bacteria and serum. The antioxidant factors showed significant reductions in SOD, CAT, and MDA levels (*p* < 0.01), the mRNA expression of anti-inflammatory factors was significantly reduced (*p* < 0.01). Histopathological examination indicated that organ tissue structures in the experimental group remained intact. Furthermore, kidney cell apoptosis and DNA damage were assessed through immunofluorescence testing, revealing significant reductions in apoptosis factor p53 and DNA damage factor γH2A.X in the experimental group (*p* < 0.01). These results indicate that the VF14355 protein has immunoprotective activity against different bacterial infections. In previous studies, Xu et al. [29] utilized the recombinant outer membrane protein OmpTS of *A. hydrophila* as a subunit vaccine to immunize and subsequently challenge bream fish (*Megalobrama amblycephala*), and found that the survival rate in the immunized group was significantly higher than that of the control group. Additionally, serum immune factors, IgM-specific antibody titers, and antibacterial enzyme activities were elevated in the immunized group. A bacterial coating test revealed a reduction in the relative abundance of *A. hydrophila* in tissues. The OmpTS subunit vaccine effectively induces an immune response in bream fish. Sun et al. [26] prepared an ExbB protein vaccine of *P. fluorescens*, and found that it could activate the non-specific immune activity in *C. auratus*, and the immune protection rates against *P. fluorescens* and *A. hydrophila* were 54% (*p* < 0.05) and 38.4% (*p* < 0.05), respectively. Therefore, this study employed more molecular methods to confirm that the VF14355 protein could resist infections from multiple bacteria, positioning it as a multivalent vaccine candidate for aquaculture. However, the immunization method was intraperitoneal injection, which increased the cost of immunization in aquaculture. Thus, it is necessary to conduct subsequent research on nanoparticle encapsulation to adopt an oral immunization approach.

Egg yolk (IgY) antibodies offer several advantages, including high antigen specificity, low cost, wide availability, simple preparation, lack of toxic side effects, and alignment with animal welfare principles [30,31]. Consequently, they represent a green and safe alternative to antibiotics. IgY has been extensively utilized in health research [32] as both a therapeutic and a diagnostic tool [33]. In the current study, *C. auratus* were immunized with VF14355 IgY and subsequently challenged with *V. fluvialis* and *A. hydrophila*. The IgY immunization enhanced the levels of immune factors, and Western blotting confirmed the specificity of IgY with a titer reaching 1:102,400. The VF14355 IgY immunization provided a significant protective effect against bacterial infections (*p* < 0.01). A bacterial coating test indicated that IgY immunization significantly reduced the number of bacteria in the kidneys (*p* < 0.01); additionally, the phagocytosis index and phagocytosis percentage of white blood cells increased (*p* < 0.01). IgY immunization elevated the levels of immune factors such as ACP, AKP, and LZM (*p* < 0.01). Furthermore, the expression of inflammatory and antioxidant factors was down-regulated (*p* < 0.01) to reduce an inflammatory and antioxidant response, respectively. Histopathological examination revealed that IgY immunization preserved the integrity of visceral tissue structure, and immunofluorescence experiments demonstrated that IgY immunization effectively reduced apoptosis and DNA damage induced in the kidney cells of *C. auratus* (*p* < 0.01). These results indicate that the VF14355 IgY has immunoprotective activity against different bacterial infections. In previous studies, Zhang et al. [34] administered anti-*Streptococcus agalactiae* IgY to tilapia, followed by a challenge with *S. agalactiae*, the results demonstrated that the IgY reduced the proportion of *Streptococcus* and enhanced the diversity of intestinal flora. Histopathological analyses revealed that the IgY mitigated tissue damage. Immunofluorescence experiments indicated that the IgY reduced apoptosis in intestinal epithelial cells. The expression of antioxidant and inflammatory factors showed a reduction. Collectively, the IgY of *S. agalactiae* has immune activity. Liu et al. [35] prepared IgY from *P. fluorescens* OMPs (PF1380 and ExbB), and the passive immunization to *C. auratus* showed that the two IgY had an immune protection rate, down-regulated the expression of antioxidant-related factors and inflammation-related genes, maintained the integrity of visceral tissue structure, and reduced apoptosis and damage of tissue cells in relation to *P. fluorescens* and *A. hydrophila* infections. Furthermore, the IgY antibodies of OMPs (OmpAII, OmpW, P5, and Slp) of *A. hydrophila* were prepared, and passive immunization to fish showed that the passive (*A. hydrophila*) and passive cross-protective (*P. fluorescens*) abilities of OmpW and Slp were higher than those of OmpAII and P5 [36]. Additionally, the passive immunization of fish with IgY antibody enables them to immediately acquire the ability to resist bacterial infections, and IgY can be economically prepared in large quantities from egg yolk. Further, IgY had application value in aquaculture. Therefore, this study indicates that the IgY of VF14355 can combat infections from various bacterial strains, suggesting its potential as a multivalent vaccine candidate for aquaculture.

DNA vaccines, referred to as nucleic acid vaccines or gene vaccines, involve the direct injection of a recombinant eukaryotic expression vector that encodes a specific protein antigen into an animal’s body, a process that enables the expression of the foreign gene in vivo and leads to the production of the antigen that activates the body’s immune system, thereby inducing specific humoral and cellular immune responses [37,38,39]. DNA vaccines have garnered significant attention in aquaculture, particularly through their combination with plasmids that carry pathogen-specific antigens to enhance protective immunity against various fish pathogenic diseases [40]. In the current study, the VF14355 DNA vaccine was administered to immunize *C. auratus* against challenges posed by *V. fluvialis* and *A. hydrophila*, and the results indicated that the protection rate in the VF14355-immunized group was significantly higher than that in the control group (*p* < 0.01). Furthermore, the bacterial load in the kidneys of the immunized group was significantly reduced (*p* < 0.01), while the ELISA results revealed an in vitro interaction between the bacteria and *C. auratus* serum. Notably, the plasma phagocytosis index and phagocytosis percentage and serum immune factors in the immunized group were significantly elevated (*p* < 0.01). Conversely, the levels of serum antioxidant factors and the mRNA expression levels of inflammatory factors were significantly decreased (*p* < 0.01). Histopathological examination revealed that the organ structures remained intact, and immunofluorescence tests demonstrated significant reductions in apoptosis factor p53 and DNA damage factor γH2A.X, suggesting that the VF14355 DNA vaccine effectively induces an immune response in *C. auratus* and provides excellent immune protection. In previous studies, Hu et al. [41] developed a DNA vaccine composed of the main viral capsid protein (MCP) and actively immunized *Largemouth bass*, finding a significant increase in specific serum IgM levels. Furthermore, the expression levels of humoral immunity markers (CD4), cellular immunity markers (MHCI-α), cytokines (IL-1β), and immune-related factors (ACP, AKP, and LZM) were increased. Additionally, following a challenge with virus, the relative survival rate of fish was 89.5%, demonstrating that the DNA vaccine effectively induces both humoral and cellular immune responses, providing protection to *Largemouth bass* against virus challenges. Similarly, Zhang et al. [42] developed the pcDNA-PK DNA vaccine targeting *Nocardia seriolae* and administered it to *Channa maculata*, the results of which indicated that, compared to the control group, the vaccinated fish exhibited increased serum enzyme activity parameters, including LZM, SOD, ACP, and AKP. Additionally, specific antibody IgM levels were elevated, and six immune-related genes (CD4, CD8α, TNFα, IL-1β, MHCIα, and MHCIIα) were up-regulated. Following a challenge with *N. seriolae*, the immunized group achieved a protection rate of 53.82%, suggesting that the pcDNA-PK DNA vaccine can significantly enhance the immune response in *C. maculata*. However, there is a risk that exogenous DNA entering the fish body may integrate into the host genome, and this study requires further evaluation of the safety of the VF14355 DNA vaccine. Addition, the VF14355 DNA vaccine requires further preparation into an oral nanoformulation to reduce immunization costs. Overall, the VF14355 DNA vaccine effectively induces an immune response, and can resist infections from multiple bacteria in *C. auratus*, suggesting its potential as a multivalent vaccine candidate for aquaculture.

## 4. Materials and Methods

### 4.1. Strains and Animals

*V. fluvialis* ATCC33809, *A. hydrophila* ATCC7966, and *Staphylococcus aureus* (*S. aureus*) ATCC6538, as well as the *V. fluvialis* outer membrane protein VF14355 (GenBank: AMF94637.1) and VF14355 DNA vaccines, were preserved in the Microbiology Laboratory of Fuyang Normal University. Twenty-week-old Leghorn laying hens were procured from Chongqing Tengxin Biotechnology Co., Ltd. (Chongqing, China), and *C. auratus* (20 ± 1.0 g) were sourced from Fuyang Aquaculture Co., Ltd. (Fuyang, China). All animal experiments were conducted in accordance with the Guide for the Care and Use of Laboratory Animals, and approval was received from the Institutional Animal Care and Use Committee of Fuyang Normal University, China (2024-03-002).

### 4.2. Preparation of IgY Antibodies

Each chicken was immunized four times with 200 μg of VF14355 protein, with an interval of 14 days between each immunization. Freund’s complete adjuvant was utilized for the initial immunization, while Freund’s incomplete adjuvant was employed for the subsequent booster immunizations. Eggs were collected each day, for a duration of 40 days, post-immunization. The yolks from the selected eggs were separated using a yolk separator, whereafter phosphate-buffered saline (PBS) at pH 7.2 was added in a 1:1 ratio by volume and mixed thoroughly. Subsequently, 3.5% powdered polyethylene glycol 6000 (PEG6000) was incorporated, and the mixture was shaken at 25 °C, at a speed of 100 r/min, for 30 min, after which the mixture was centrifuged at 10,000 r/min, and the supernatant was filtered through filter paper. An additional 8.5% PEG6000 was then added to the filtrate, mixed thoroughly, and shaken again at 25 °C, at 100 r/min, for 30 min. Following another round of centrifugation at 10,000 r/min, the supernatant was discarded, and the precipitate was dissolved in 10 mL of PBS, followed by the addition of 12% PEG6000; this mixture was shaken at 100 r/min for 30 min, allowed to stand for 10 min, and then centrifuged. The resulting precipitate was dissolved in 2 mL of PBS. Finally, the solution was placed in a dialysis bag and dialyzed with PBS at 4 °C for 36 h [43].

### 4.3. Western Blotting

Western blotting was employed to assess the specificity of IgY. *V. fluvialis* was cultured overnight at 30 °C and, following bacterial collection via centrifugation, 300 μL of sodium dodecyl sulfate (SDS) loading buffer was added; the mixture was then shaken, mixed, and boiled for 5 min. The resulting solution was loaded onto gel for SDS-PAGE, after which it was transferred to a membrane at 4 °C and 80 V for 60 min. After electrophoresis, the nitrocellulose (NC) membrane was blocked overnight at 4 °C in 5% skim milk. The membrane was washed three times with PBS solution and subsequently incubated at room temperature for 2 h with a gradient dilution of IgY (1:400–1:102,400). After washing three times with the PBS solution, the membrane was incubated with a secondary antibody (HRP-conjugated Affinipure Rabbit Anti-Chicken IgY (IgG) (H + L), diluted 1:1000) at 37 °C for 1 h. Finally, the membrane was washed three times with PBS and developed using the enhanced chemiluminescence (ECL) luminescent solution [44].

### 4.4. Interaction Detection In Vitro

The ELISA was employed to identify the interaction between antibodies and bacteria. *V. fluvialis* was cultured to an optical density (OD_600_) of 1.0, after which the bacteria were collected using centrifugation, the pellet was resuspended in physiological saline to achieve an OD_600_ of 1.0, and 200 μL per well was added to the enzyme plate. The plate was coated at 4 °C overnight and subsequently washed three times with PBS solution. Following a blocking step with 5% skimmed milk powder at 37 °C for 1.5 h, the cells were again washed three times with PBS solution. Gradient dilutions of IgY/*C. auratus* serum (1:100, 1:200, 1:400, 1:800, 1:1600, 1:3200) were then added, and the mixture was incubated at 37 °C for 1 h before washing three times with PBS solution. A secondary antibody, either HRP-conjugated Affinipure Rabbit Anti-Chicken IgY (IgG) (H + L), diluted 1:1000, or rat anti-fish IgM, diluted 1:400, was added and incubated at 37 °C for 1 h. After washing three times with the PBS solution, a chromogenic solution was added and incubated in the dark at 37 °C for 10 min, followed by the addition of a solution to terminate the reaction. The absorbance (*OD*_450_) was then immediately read using a microplate reader [44].

### 4.5. Active Protective and Active Cross-Protective Rates of VF14355

*C. auratus* were immunized with protein (2 μg/g), and the volume of the protein solution was 40 μL. Freund’s incomplete adjuvant and recombinant outer membrane protein were mixed and injected into the abdominal cavity of *C. auratus*. The *C. auratus* specimens were divided into a nature control group (NC) receiving normal saline and an experimental group receiving VF14355 protein, with 15 fish in each group. Two immunizations were administered: one on day 1 and the second on day 10; 7 days after the second immunization, the *C. auratus* were intraperitoneally challenged with *V. fluvialis* (8 × 10^8^ CFU) and *A. hydrophila* (4.0 × 10^8^ CFU), respectively. Specimens were observed and recorded for 14 days, after which immune protection rates were calculated and immune activities were evaluated [44].

### 4.6. Passive Protective and Passive Cross-Protective Rates of VF14355 IgY Antibody

The *C. auratus* specimens were divided into a control group and an experimental group, each consisting of 15 fish. The nature control group (NC) received an intraperitoneal injection of 30 μL of normal saline, while the experimental group was immunized with 30 μL of VF14355 IgY antibody. After 2 h, the challenge bacteria of *V. fluvialis* and *A. hydrophila* were set at 1 × 10^9^ CFU and 4.2 × 10^8^ CFU, respectively. Fish mortality was monitored over a period of 14 consecutive days. The relative percent survival (RPS) was calculated using the following formula: RPS (%) = (1 − [% mortality rate of experimental group/% mortality rate of control group]) × 100. Significant differences between the experimental and control groups were analyzed using SPSS 19.0 software [35].

### 4.7. Active Protective and Active Cross-Protective Rates of VF14355 DNA Vaccine

The constructed VF14355-pcDNA3.1 recombinant strain was expanded and cultured, followed by plasmid extraction and filtration through a 0.22 μm filter to prepare the VF14355 protein DNA vaccine. *C. auratus* were immunized with the plasmid (1 μg/g), and the volume of the plasmid solution was 40 μL. *C. auratus* were divided into a control group (receiving normal saline) and an experimental group, each consisting of 15 fish, and immunized via an intraperitoneal injection. Two immunizations were conducted, on the 1st and 10th days. Seven days after the second immunization, the challenge bacteria of *V. fluvialis* and *A. hydrophila* were determined to be 8 × 10^8^ CFU and 4.0 × 10^8^ CFU, respectively. Observations and recordings were made over a period of 14 days, after which the immune protection rate was calculated and immune activity was evaluated [45].

### 4.8. Immune Factor Detection

Seven days after the second vaccination of *C. auratus* with VF14355 protein and its DNA vaccine, and ten days after the third vaccination of laying hens with VF14355 protein, blood was taken from the tail vein of *C. auratus* and from the lower wing vein of laying hens, and serum was obtained via centrifugation. The levels of immune factors ACP, AKP, and LZM were evaluated according to the instructions of the detection kit (Jiancheng Institute of Biotechnology Co., Ltd., Nanjing, China) [36].

### 4.9. Kidney Bacterial Content

Two days after the pathogenic bacteria challenge, kidney tissue was aseptically removed from *C. auratus* on a clean workbench and homogenized in a homogenizer. Subsequently, 400 μL of physiological saline was added to prepare a homogenate. Then, 200 μL of the mixed solution was spread on an LB solid medium. The plates were incubated upright at 30 °C for 1 h, then inverted and incubated overnight. Finally, photographs were taken and the number of colonies was counted [36].

### 4.10. Leukocyte Phagocytosis Analysis

Two days after the pathogenic bacteria challenge, and ten days after the third VF14355 protein immunization of the laying hens, plasma was taken from each *C. auratus* via the tail vein and laying hen via a lower wing vein, and Staphylococcus aureus was inactivated with 1% formaldehyde saline at 80 °C for 90 min, after which the bacteria were adjusted to an OD_600_ = 0.6 with saline. Thereafter, 0.2 mL of *C. auratus*/chicken plasma was mixed with 0.2 mL of inactivated *S. aureus* and bathed in water at 25 °C for 60 min. Blood smears were prepared with 10 μL mixture and fixed with methanol. After methanol volatilization, a rapid Giemsa staining kit (Sangon Biotechnology Co., Ltd., Shanghai, China) was used for staining. Phagocytic cells were counted under a microscope, and the calculation method was as follows: phagocytic percentage (*PP*%) = number of phagocytic cells involved in 100 phagocytic cells/100 × 100%, and phagocytic index (*PI*%) = number of bacteria in phagocytic cells/number of cells involved in phagocytic cells × 100% [46].

### 4.11. Antioxidant Factor Analysis

Two days following the pathogenic bacteria challenge, blood samples were collected from the tail veins of the *C. auratus* specimens and subsequently centrifuged to obtain serum. The antioxidant factor levels, specifically those of SOD, CAT, and MDA, were assessed in accordance with the instructions provided in the detection kit (Sangon Biotechnology Co., Ltd., Shanghai, China) [43].

### 4.12. mRNA Expression of Inflammatory Factors

The real-time quantitative PCR (qRT-PCR) method was employed to assess the expression levels of inflammatory factor mRNA. On the second day post-challenge, kidney and spleen tissues from *C. auratus* were collected and placed in a mortar. Liquid nitrogen was added, and the tissues were ground using a pestle. RNA was extracted following the protocols provided in the RNA extraction kit (Sangon Biotech Co., Ltd., Shanghai, China). Subsequently, cDNA was synthesized according to the instructions of the RNA extraction kit (Takara Biotechnology Co., Ltd., Beijing, China), utilizing a two-step reaction program (95 °C for 30 s; 95 °C for 15 s; 60 °C for 30 s; repeated for 40 cycles). For qRT-PCR, the SYBR^®^ Green Premix kit (Takar Biotechnology Co., Ltd., Beijing, China) and synthetic primers (Table 1) were utilized [36].

### 4.13. Histopathological Analysis

Two days after *C. auratus* specimens were exposed to pathogenic bacteria, their kidney, spleen, and small intestine tissues were fixed in Davidson’s fixative for over 18 h. The tissues were then transferred to a 10% formaldehyde solution for more than 24 h, and subsequently subjected to dehydration in gradient ethanol solutions (80%, 90%, 95%, and 100%) for 40 min, 20 min, 15 min, and 10 min, respectively. Following dehydration, the tissues were treated with xylene for transparency, using a 1:1 mixture of absolute ethanol and xylene, followed by treatments in xylene I and xylene II for 30 min each. The samples were then placed in paraffin, immersed in a 1:1 mixture of paraffin and xylene, and subjected to paraffin I, II, and III treatments for 30 min each. After immersion in wax, the tissues were placed into molds to solidify. The solidified tissues were then cut into 5 μm sections using a paraffin microtome and baked at 37 °C for 24 h, after which the dried sections were stained with hematoxylin and eosin (H&E). To prepare the sections for staining, they were deparaffinized in xylene I and II solutions for 10 min, rehydrated in 100%, 95%, 80%, and 70% ethanol gradients for 5 min each, stained with hematoxylin for 5 min, rinsed with tap water, and differentiated in 1% hydrochloric acid alcohol. After a brief rinse with tap water, the sections were stained with eosin for 30 min, dehydrated through an ethanol gradient (85%, 95%, and 95%) for 10 s, dehydrated in absolute ethanol for 3 min, cleared with xylene for 5 min, and finally sealed with neutral resin before being observed under a microscope [45].

### 4.14. Renal Immunofluorescence Analysis

The prepared kidney slices were dewaxed three times in xylene, followed by rehydration in ethanol, using a decreasing concentration gradient (100%, 100%, 95%, 80%, 50%), and subsequently washed three times with PBS. The slices were then soaked in 1% Triton X-100 for 30 min, boiled in carbonate buffer (CBS), and allowed to cool naturally, after which they were immersed in 3% hydrogen peroxide (H_2_O_2_) for 3 min and washed again with PBS three times. An immunohistochemistry pen was used to outline the periphery of the tissue, and a 50 μL (5%) fetal bovine serum albumin (BSA) blocking solution was applied within the outlined area, followed by blocking at room temperature for 1.5 h. After washing with PBS, monoclonal antibodies against p53 or γH2A.X (1:500) were added to the tissue and incubated overnight at 4 °C. Following another round of PBS washing, a 1:500 diluted secondary antibody solution (Donkey anti-Rabbit IgG) was applied and incubated at 37 °C for 1 h. The nuclei were stained with DAPI and observed under a fluorescence microscope to capture images. Then, the images were analyzed for fluorescence intensity with ImageJ 1.54g software. Finally, the immunofluorescence value of p53 and γH2A.X were obtained to assess their expression in the kidney [36].

### 4.15. Statistical Analysis

All the experimental data were expressed as the mean ±SD. The significant difference from the respective control in all experiments was assessed by a one-way analysis of variance (ANOVA) using SPSS (IBM Corporation, Chicago, IL, USA). Values of *p* < 0.05 were considered statistically significant [35].

## 5. Conclusions

In summary, this study was conducted with the aim of assessing VF14355 protein, DNA, and IgY vaccines in *C. auratus*, and these three vaccines were found to confer significant protection rates against bacterial infection, reduce the number of kidney bacteria, enhance leukocyte phagocytosis activity, and increase the immune factor expression levels of *C. auratus.* Furthermore, fish antibodies recognized the bacteria in vitro. Moreover, the three vaccines improved anti-inflammatory and antioxidant activities, protected the integrity of visceral tissue, and alleviated apoptosis and DNA damage in visceral tissue cells due to bacterial infection in *C. auratus*. Therefore, the three investigated vaccines showed immune protection against different bacterial infections and are potential candidates for polyvalent vaccines in fish.

## Figures and Tables

**Table 1:** Primers used for the qRT-PCR.

Gene	NCBI Number	Forward Primer (5–3′)	Reverse Primer (5–3′)
*il-6*	XM_026289280.1	TCTCCTCAGACCCTCAGACG	CGTTTGGTCCCGTGTTTGAC
*il-8*	XM_026267284.1	GGAGTGCAGGCCACTGTTAG	ATCAGAAGCATGAAGGCGGA
*il-1β*	AJ249136.1	TTCAGGAAAGAGACGGGCAC	GTCAGTTGGCACCTGGATCA
*tnf-α*	EU069817.1	GGGCCACATCGTGATTCGTA	GCCTCCAGTGTAGCATGTGT
*gapdh*	XM_026284269.1	GATTTCAACGGGGATGTGCG	TCACACACACGGTTGCTGTA

## Data Availability

The data is contained within the article and Supplementary Materials.

## Supplementary Materials

The following supporting information can be downloaded at: https://www.mdpi.com/article/10.3390/ijms26073379/s1.
