# Supplementary material for: Evaluation of the Multivalent Immunoprotective Effects of Protein, DNA, and IgY Vaccines Against Vibrio fluvialis Outer Membrane Protein VF14355 in Carassius auratus
Source: Int J Mol Sci. 2025 Apr 4;26(7):3379. doi: 10.3390/ijms26073379 (PMC11989368; doi:10.3390/ijms26073379)
Supplement: Supplementary file 1 [file ijms-26-03379-s001.zip › Supplementary Figure S1.pdf]

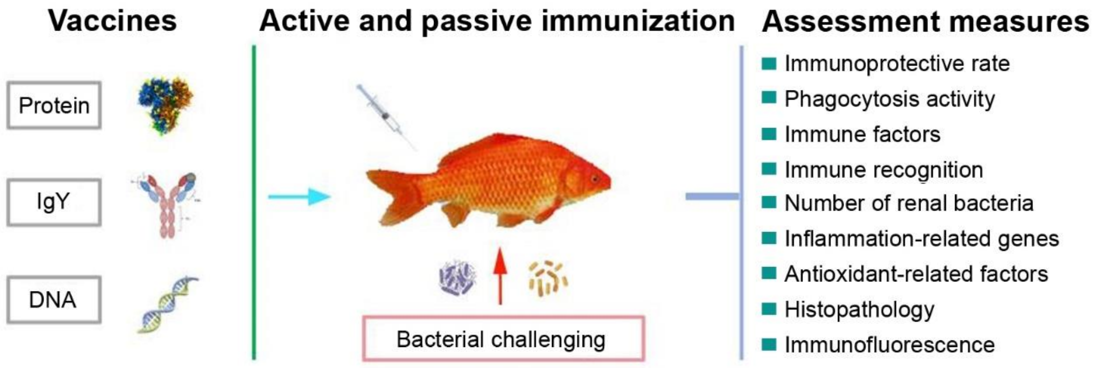

Supplementary Figure S1. Experimental process. The protein, DNA, and IgY vaccines of VF14355 protein of *V. fluvialis* were administered to *C. auratus* subsequently challenged with *V. fluvialis* and *A. hydrophila*. The immune efficacy of the three vaccines was evaluated through the indexes of immunoprotective rate, phagocytosis activity, immune factors, immune recognition, number of renal bacteria, inflammation-related genes, antioxidant-related factors, histopathology, and immunofluorescence.
