# Supplementary material for: Evaluation of the Multivalent Immunoprotective Effects of Protein, DNA, and IgY Vaccines Against Vibrio fluvialis Outer Membrane Protein VF14355 in Carassius auratus
Source: Int J Mol Sci. 2025 Apr 4;26(7):3379. doi: 10.3390/ijms26073379 (PMC11989368; doi:10.3390/ijms26073379)
Supplement: Supplementary file 1 [file ijms-26-03379-s001.zip › Supplementary Figure S2.pdf]

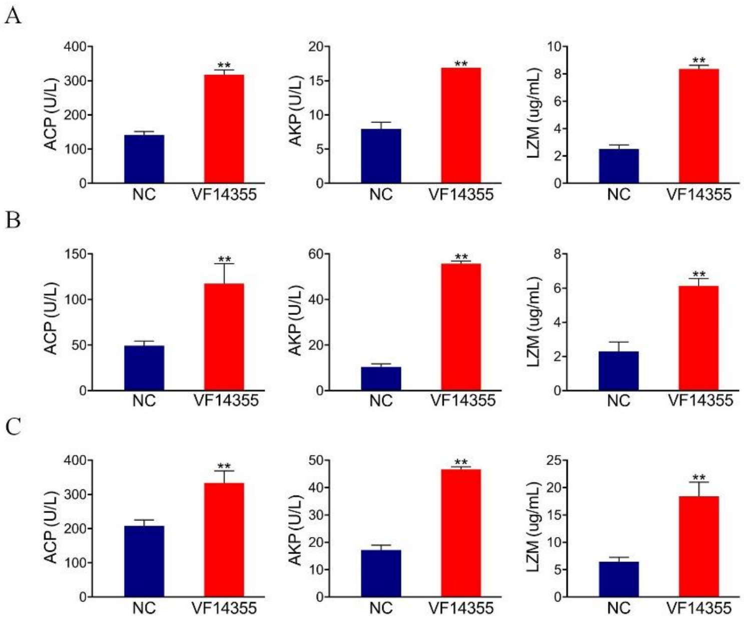

Supplementary Figure S2. Expression of immune factors. A, B and C represent the protein, IgY and DNA vaccines of VF14355. Compared with control group, $^{**}p < 0.01$.
