# Supplementary figures and images for: Evaluation of the Multivalent Immunoprotective Effects of Protein, DNA, and IgY Vaccines Against Vibrio fluvialis Outer Membrane Protein VF14355 in Carassius auratus
Source: Int J Mol Sci. 2025 Apr 4;26(7):3379. doi: 10.3390/ijms26073379 (PMC11989368; doi:10.3390/ijms26073379)

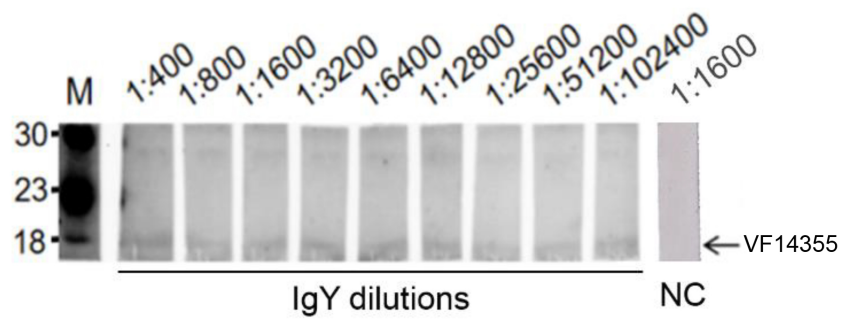

**Supplementary Figure S3.** The titer and specificity of VF14355 IgY antibody.

Supplement: Supplementary file 1 [file ijms-26-03379-s001.zip › Supplementary Figure S3.pdf]
